# Disposable Device for Bacterial Vaginosis Detection

**DOI:** 10.1021/acsmeasuresciau.3c00007

**Published:** 2023-06-17

**Authors:** Mariana D. Avila-Huerta, Karina Leyva-Hidalgo, Karen Cortés-Sarabia, Ana K. Estrada-Moreno, Amalia Vences-Velázquez, Eden Morales-Narváez

**Affiliations:** † Centro de Investigaciones en Óptica, A. C., Loma del Bosque 115, Lomas del Campestre, León 37150, Guanajuato, Mexico; ‡ Facultad de Ciencias Químico Biológicas, Universidad Autónoma de Guerrero, Chilpancingo 39070, Guerrero, Mexico; § Biophotonic Nanosensors Laboratory, Centro de Física Aplicada y Tecnología Avanzada (CFATA), Universidad Nacional Autónoma de México (UNAM), Querétaro 76230, Mexico

**Keywords:** rapid diagnostics, infectious diseases, biophotonics, fluorescence resonance energy transfer, immunoassay

## Abstract

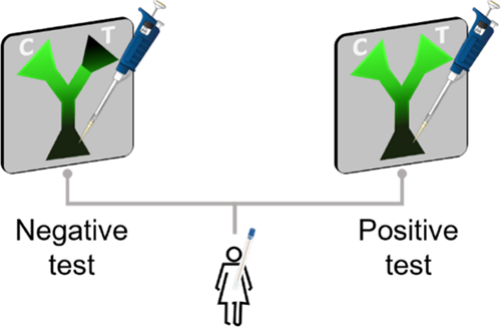

Due to the increasing demand for clinical testing of infectious diseases at the point-of-care, the global market claims alternatives for rapid diagnosis tools such as disposable biosensors, avoiding the need for specialized laboratories and skilled personnel. Bacterial vaginosis (BV) is an infectious disease that commonly affects reproductive-age women and predisposes the infection of sexually transmitted diseases. Especially in asymptomatic cases, BV can lead to pelvic inflammatory conditions, postpartum endometritis, and preterm labor. Conventionally, BV diagnosis involves the microscopic analysis of vaginal swab samples; it thus requires highly trained personnel. In response, we report a novel microfluidic paper-based analytical device for BV diagnosis. Sialidase, a biomarker overexpressed in BV, was detected by exploiting an immunosensing mechanism previously discovered by our team. This technology employs a graphene oxide-coated surface as a quencher of fluorescence; the fluorescence of the immunoprobes that do not experiment immunoreactions (antibody–antigen) are deactivated by graphene oxide via non-radiative energy transfer, whereas those immunoprobes undergoing immunoreactions preserve their photoluminescence due to the distance and the low affinity between the immunocomplex and the graphene oxide-coated surface. Our paper-based test was typically carried out within 20 min, and the sample volume was 6 μL. Besides, it was tested with 14 vaginal swabs specimens to discriminate clinical samples of women with normal microbiota from those with BV. Our disposable device represents a new tool to prevent the consequences of BV.

## Introduction

Disposable biosensors are affordable and easy-to-use devices for a single measurement, which are now integrated into our daily life, for example, in pregnancy or fertility tests and wearable blood glucometers.^1^ Microfluidic paper-based analytical devices (μPADs) have a wide range of applications as disposable biosensors. These devices are promising tools for medical diagnostics and environmental monitoring and can be used at the point-of care (POC).^2,3^ μPADS are easy to manufacture and low-cost. In fact, μPADS are usually fabricated using printing techniques or drop-on-delivery mechanisms (e.g., hydrophobic printing, flexographic printing, and selective laser sintering, among others),^4^ avoiding the need of complex microfluidic components such as pumps and valves.^3,4^ The most representative example of μPADS is the lateral flow immunoassay. Such a test determines the presence of a biomarker using different antibodies to capture and detect the analyte in a paper strip format.^1^ In general, μPADS have been engineered to determine different analytes using optical and electrochemical transduction systems, for instance, glucose, uric acid, antibody IgG, cancer biomarkers, hepatitis C virus, Zika viral gene markers, *Escherichia coli*, etc.^5−10^

Bacterial vaginosis (BV) is an infectious disease induced by an alteration in the vaginal microbiota and commonly affects reproductive-age women. Generally, the symptoms of BV include moderate white-grayish vaginal secretion after sexual intercourse, fishy odor, vaginal discharge, and in some cases dysuria and dyspareunia. However, this infectious disease may occur as an asymptomatic condition, and this condition may eventually lead to negative impacts in reproductive health, such as postpartum endometritis, pelvic inflammatory disease, and predisposition to sexually transmitted diseases, including those caused by *Chlamydia trachomatis*, *Neisseria gonorrhoeae*, human papillomavirus, and human immunodeficiency virus.^11−13^ Typically, clinical diagnosis of BV is based on the evaluation of the Amsel^14^ or Nugent^15^ criteria, which are performed by highly trained personnel.^11,16^ However, patients may be misdiagnosed because of the lack of specialized diagnostic tools or the strict application of the diagnostic criteria of the respective clinical indicator tests (white or grayish vaginal discharge, vaginal pH > 3.5, amine production, and the presence of clue cells).^11,12^ Since BV triggers an alteration of the microbiota, a diverse community of anaerobic bacteria are involved in this condition, such as *Prevotella*, *Bacteroides*, *Mobiluncus*, and *Gardnerella*, and all of them produce hydrolytic enzymes such as sialidase (SLD). Therefore, SLD can be employed as a biomarker of BV.^11,12^ A common technique for the detection of SLD in vaginal swabs is based on the enzymatic hydrolysis of methoxyphenyl acetyl muramic acid via the catalytic character of SLD, thereby initiating the formation of methoxyphenol, which is then measured. Usually, in this technique, methoxyphenol concentrations greater than 5.1 nM are considered as BV positive. As far as we know, only one commercially available test targeting SLD exists: OSOM BVBLUE (Sekisui Diagnostics, Burlington, MA).^17^ Nevertheless, this test offers a multistep qualitative colorimetric result for the determination of SLD, which eventually leads to different levels of specificity and sensitivity.^11^ In this context, our collaborative team engineered a monoclonal antibody that recognizes SLD with high specificity.^18^

**Figure 1:**
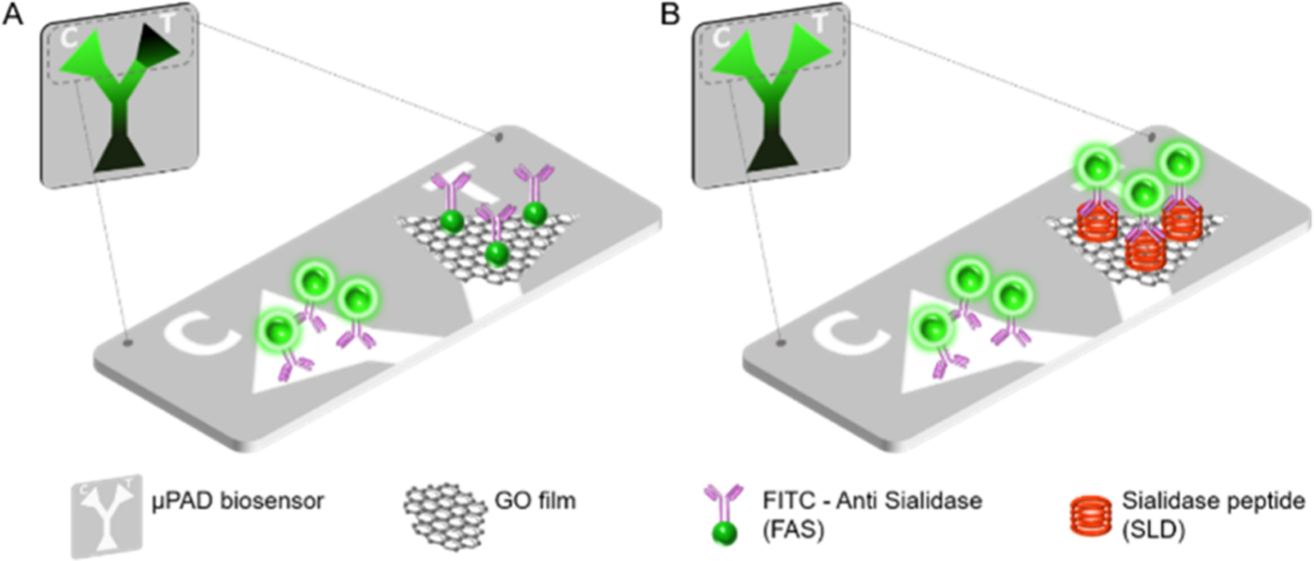
Schematic representation of the disposable device for BV diagnosis and its overall biosensing mechanism. In the control area (C), FAS preserves its conventional fluorescent intensity, due to the lack of a reagent (such as GO) that can modify the FAS fluorescence emission state. (A) In the absence of immunoreactions in the assay, fluorescence intensity of FAS is quenched in the test zone (T) by the GO film via non-radiative energy transfer. (B) When FAS experiments immunoreactions in the assay, the fluorescent intensity of FAS is preserved according to the analyte concentration, because of the distance between FITC and GO (>10 nm) and the low affinity between the immunocomplex and the GO film.

Herein, as a potential diagnosis tool, we developed a wax-printed μPAD to detect SLD, even in clinical samples. Considering the capabilities of graphene oxide (GO) to quench fluorescence in a highly efficient way,^11,19−22^ our research group has developed a high-throughput biosensing system that consists of photoluminescent bioprobes and 96-microwell plates modified with GO. Using non-radiative energy transfer, GO-coated microwells deactivate the fluorescence of the bioprobes that are not establishing immunoreactions, as the distance between the bioprobes and GO (<10 nm) allows for the dipole–dipole interaction.^23^ On the contrary, fluorescent biorecognition probes showing immunoreactions preserve their photoluminescence because of the distance (>10 nm) and the low affinity between the immunocomplex (biorecognition probe—analyte) and the GO-coated surface.^11,19−21^ The distance between the FITC (from FAS) and the GO (in the surface of the membrane) can be estimated considering the size of the anti-SLD antibody plus the size of SLD, which is 8.4 + 4 nm, respectively, that is, c.a. 12.4 nm (see Figure S1 in the Supporting Information).^24,25^ We are taking advantage of this wash-free technology to develop a disposable device for VB diagnosis. Since other tests for BV diagnosis are based on catalytic reactions,^11^ our approach represents the first paper-based immunoassay for BV detection. The device comprises a wax pattern printed onto a nitrocellulose membrane. The strip displays a Y-shaped pattern, including three zones of interest: (i) the entrance, where the probe and the sample are drop-casted; (ii) the control zone, where the biorecognition probe, composed by a monoclonal anti-SLD antibody conjugated with FITC fluorophore (FAS), will preserve its conventional fluorescence since bare nitrocellulose does not strongly affect the fluorescent emission of the biorecognition probe, and (iii) the test zone, which is the area coated with a GO film. By means of the aforementioned non-radiative energy transfer, the GO film from the test zone deactivates the fluorescence of those FAS that are not experimenting immunoreactions, whereas those FAS experimenting immunoreactions preserve their photoluminescence. Hence, the photoluminescence exhibited in the test zone by FAS is proportional to the analyte concentration, as illustrated in Figure 1.

## Materials and Methods

### Materials and Equipment

Nitrocellulose membrane (1UN18ER100025NT) was purchased from Sartorius (Göttingen, Germany). Aqueous suspension of single-layer GO (S1319112702) was purchased from Global Graphene Group (Dayton, OH, USA). According to the supplier, this 2D material displays an average lateral size around 50 nm and its C/O ratio is around 1. Tween 20 (P9416-100ML) and phosphate buffer saline (PBS) tablets (P4417-100TAB) were purchased from Sigma-Aldrich (Saint Louis, MI, USA). FITC Conjugation Kit Fast-Lightning-Link (ab188285) was purchased from Abcam (Cambridge, UK). According to previously reported procedures,^11,18^ anti-SLD monoclonal antibody and SLD peptide were produced by our team at the Universidad Autónoma de Guerrero (Chilpancingo, Guerrero, Mexico). Vaginal swab samples were collected by “Servicio de Diagnóstico Integral en la detección Oportuna del Cancer Cérvico Uterino” at Universidad Autónoma de Guerrero (Chilpancingo, Guerrero, Mexico). Signed consent was obtained from those women who participated in this research. Wax patterns were printed using a ColorQube 8085 from Xerox (Stanford, CT, USA). A hot plate StableTemp from Cole-parmer (Vernon Hills, IL, US) was used to heat and fabricate the paper-based devices. All the fluorescent micrographs were acquired through a Cytation 5 multimodal reader from BioTek (Winooski, VT, USA).

### Fabrication of the Paper-Based Device

The fabrication of the proposed μPAD biosensors is straightforward and only requires two steps: (i) print the wax pattern onto the nitrocellulose membrane; and (ii) heat the device using a hot plate at 150 °C for 90 s (in order to form the required hydrophobic barriers). Once the device reaches the room temperature, this is ready to use. Due to the absorbent capabilities of nitrocellulose,^26^ it is relatively easy to coat this substrate with GO. To this end, GO was diluted using ultrapure water supplemented with Tween 20 at 0.05%. Deposition of GO was carried out by drop-casting the GO suspension (concentrated at 500 μg mL^–1^) onto the test zone and heating the device at 45 °C to speed up the water evaporation in which the GO was suspended. Up to three GO deposition (5 μL of GO, each deposition) steps were implemented and evaluated. Between each stage of GO deposition, it is necessary to wait for the device to dry (4 min) and then drop-cast the GO suspension once again.

### Image Analysis

All images were analyzed using ImageJ software (version 1.53t, August 24, 2022). The areas of interest for the analysis are: (i) control zone covered by the sample, (ii) test zone covered by the sample, and (iii) clear nitrocellulose, as a background section (see Figure S8 in the Supporting Information). The fluorescence quenching ratio (*Q*) was calculated according to eq 1
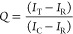
1where *I*_T_ is the average pixel intensity in the test area, *I*_C_ is the average pixel intensity in the control area, and *I*_R_ is the average pixel intensity of the background (nitrocellulose).

### BV Test

All the samples were tested in triplicate. FAS and the sample to be analyzed were mixed and incubated in a microtube during 10 min using a 1:1 relationship. The final concentration of FAS was 80 μg mL^–1^. 6 μL of this mix was then added to the entrance of the device to reach the control and test zones. After 20 min, the respective image was recorded using a Cytation 5 imager from BioTek (excitation wavelength: 469/35 nm, emission filter: 525/39 nm). Eventually, the resultant images were analyzed using the aforementioned method, and the respective fluorescence quenching ratio was calculated using eq 1.

### Vaginal Swab Sample Preparation and Analysis

Clinical samples were collected by our team at Universidad Autónoma de Guerrero (Guerrero, México). All subjects signed an informed consent based on the Helsinki declaration (2013). The samples were previously analyzed by the Amsel and Nugent criteria to compare our results with standard diagnostic methods (see Tables S2 and S3 in the Supporting Information). To determine the optimal dilution factor to be employed with real samples, two vaginal swab samples were tested, one of them from a patient with normal microbiota (NM) and the other one from a patient undergoing VB. Both samples were diluted in PBST (PBS supplemented with Tween 20 at 0.5% v/v) in a dilution factor range from 1:4 to 1:48 to determine a robust statistical difference between both samples in terms of the photoluminescent response of the developed biosensing device. Figure S10, in the Supporting Information, shows the corresponding graph. Table S4 summarizes the resultant *p*-value of the *t*-test to estimate the statistical difference between the analyzed samples. 1:32 dilution factor exhibited the smallest *p*-value (*p* = 0.0141), which corresponds to the most significant difference between the NM and the BV sample. The concentration of SLD contained in the clinical samples was estimated by interpolating the corresponding *Q* value (see eq 1) in the resulting calibration curve and multiplying by a factor of 32 (given the aforementioned optimal dilution factor).

## Results and Discussion

### Design and Fluidic Performance of the Disposable Device

To design the hydrophobic patterns, there are several parameters to consider such as the width and length of the channels, morphology of the circuit and its specific parts, as well as the volume required to fill the circuit without fluid leakage. A couple of multichannel designs were explored to determine the appropriate width channel. See Figure S2 in the Supporting Information. Each multichannel device consisted of five different width channels connected to a circular zone emerging from the center of other circular area. The widths of the channels were 0.6, 0.8, 1, 1.3, 1.5, 2, 2.5, and 3 mm, and the diameter of the circular areas were 2 and 6 mm, respectively. These multichannel devices were tested with a volume of 10 μL of anthocyanin-dyed water to observe the flow of the sample in the microfluidic circuit. In both devices, the liquid samples could not flow until the final area in the thinner channels (0.6 and 1 mm, respectively). In the wider channels with circles of 6 mm, the sample could not fill the whole area. Only in those channels between 1.3 and 1.5 mm connected to the 2 mm diameter circular area, the sample covered the interest area completely; however, some outflows were observed (see Figure S2D in the Supporting Information). Given this microfluidic performance in the multichannel devices, we decided to use a channel width of 1.6 mm (slightly wider than the 1.5 mm tested) and circular areas with a diameter of around 3 mm to avoid outflows.

The first version of our paper-based device design is shown in Figure S3 in the Supporting Information, where the entrance, control, and test zones exhibit a circular shape. From this design, some morphological modifications were made to improve the flow of the circuit and to avoid fluid leakage. Since the liquid sample contains the molecule to be detected by the disposable device, it is very important to reach the control and test zones of the microfluidic circuit, but at the same time, outflows should be avoided. To this end, FAS was diluted in PBST (PBS supplemented with Tween 20) to modify its surface tension, thereby helping the fluid to cover a larger area in the circuit. The concentration of the employed Tween 20, particularly ranging from 0.1 to 0.5% (v/v), was optimized by evaluating the area of the circuit covered by the fluid. Figure S4, in the Supporting Information, depicts that FAS diluted in PBS supplemented with Tween 20 at 0.5% (v/v) covered the largest area. Hence, this PBST composition was selected as optimal. Table S1, included in the Supporting Information, summarizes all the changes made to achieve an optimal microfluidic design, which has no outflows and covers the largest area of the microfluidic circuit. Figure S5, in the Supporting Information, shows the micrographs of the assessed designs, whose evaluation is summarized in Table S1. Figure S5G, in the Supporting Information, depicts a micrograph of the design of the disposable device with the best performance (without outflows and the largest area covered in the circuit). Figure S6 shows the dimensions and specifications of the optimal design: the length of the trunk/branches of the Y shape was 4 mm, the width of the channels was 1.6 mm, and the shape of the entrance, control, and test zones was a trapeze with a long base of 5 mm.

### Optimization of the Fluorescence Quenching

To evaluate the efficiency of the non-radiative energy transfer occurring between the GO film (acceptor) and the FAS (donor) in the disposable device, the GO film was formed in the test area of the proposed device. Several concentrations of FAS (from 40 to 100 ng mL^–1^) were also assessed. Figure S7 shows the analysis of the resulting fluorescence quenching provoked by GO concentrated at 500 μg mL^–1^ with one, two, and three depositions of GO. Using three depositions of GO, the device reached the higher quenching ratio in the test zone which, considering eq 1, ranges from 0.15 to 0.55. The optimal concentration of the bioprobe was [FAS] = 80 μg mL^–1^, which exhibited a similar fluorescence intensity in the control zone when compared with the highest concentration of FAS (100 μg mL^–1^) (see Figure S7 in the Supporting Information). Besides, in the test zone [FAS] = 80 μg mL^–1^ showed the maximum (optimal) quenching ratio (*Q*) using three depositions of GO, which is around 0.15 units (see Figure S7C in the Supporting Information).

### Biosensing Performance

**Figure 2:**
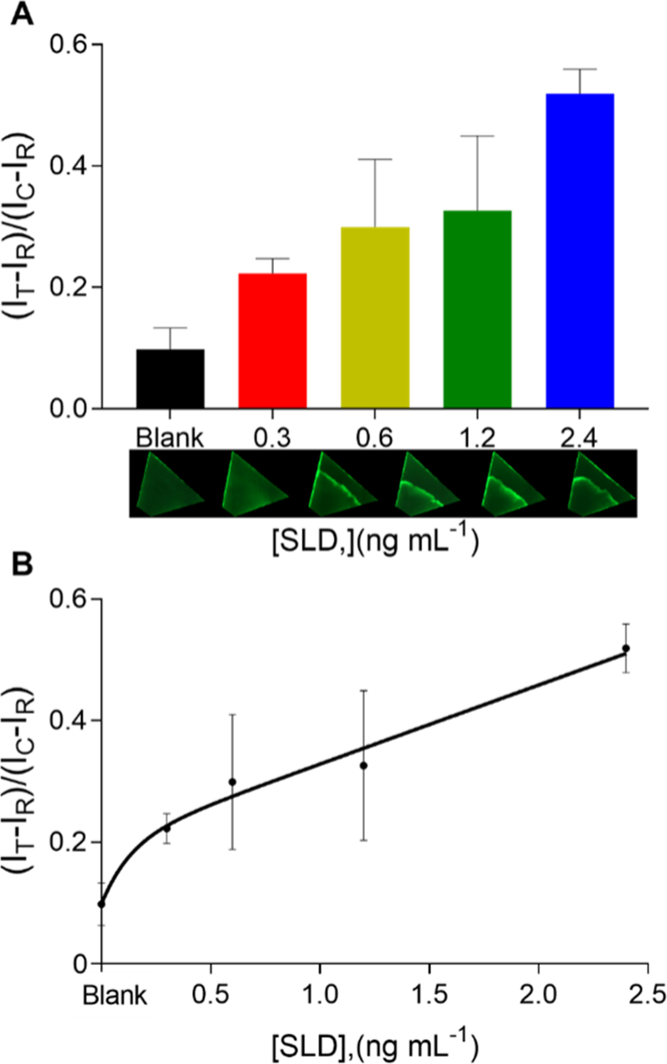
Analytical behavior of the disposable device. (A) Bar chart of the fluorescence quenching ratio, *Q*. Images below the *x* axis show the fluorescence in the test zone of the respective disposable device. (B) The resulting calibration curve. Error bars represent the standard deviation of three parallel experiments.

As discussed before, the design of the microfluidic circuit was optimized to ensure that (i) the sample reached the zones of interests (control and test zone, respectively) and (ii) the fluorescence was optimally quenched via non-radiative energy transfer between FAS (donor) and GO film (acceptor). We then proceeded to verify whether the biosensing mechanism previously developed using polystyrene microwell plates could be transferred into the proposed paper-based device. To this end, we drop-casted different samples (containing the analyte at different concentrations) in the microfluidic device, and we successfully noted that the fluorescence intensity was proportional to the analyte concentration. A calibration curve was then performed by analyzing standard samples of SLD at different concentrations, particularly from 0.3 to 4.8 ng mL^–1^ (see Figure S9). Figure 2A depicts the analytical performance of the device, which fits the two-phase exponential decay equation, where the quenching ratio (*Q*) is inversely proportional to the analyte concentration; that is, the higher the analyte concentration, the lower the *Q* ratio. In fact, in the test zone of each device, it can be observed that the higher the analyte concentration, the higher the fluorescence intensity until reaching a saturation state, in particular at those concentrations higher than 2.4 ng mL^–1^ (see Figure S9 in the Supporting Information). Figure 2B shows the resulting calibration curve. A limit of detection of 0.2 ng mL^–1^ was obtained by interpolating the mean of the blank plus three times its standard deviation in the respective calibration curve. Besides, in terms of the coefficient of variation (CV), the precision displayed by three devices measuring the same concentration ranges from 4.91 to 14.69% (see Table S5 in Supporting Information), which depicts the corresponding CVs exhibited by each measured concentration. A higher variability was observed in those samples concentrated at 0.6 ng mL^–1^, with a CV of 14.69%, whereas a lower variability was observed in the blank sample, with a CV of 4.91%. It is worth mentioning that this precision is acceptable in immunoassays.^27,28^

Prompted by these results, we tested the behavior of our disposable device with clinical samples by analyzing 14 vaginal swabs. According to Tables S2 and S3, included in the Supporting Information, the clinical samples were previously studied and determined as either BV positive or NM via the Amsel and Nugent criteria.^11,14,16^ In the Amsel criteria, the presence of at least three criteria (white or grayish vaginal discharge, vaginal pH > 3.5, amine production, and the presence of “clue cells”) is considered as a BV-positive case. The Nugent score is a standardized scored system, which is based on the classification of Gram-positive rods and lactobacilli (i.e., normal flora) and Gram-negative or Gram-variable morphotypes (BV flora). A score is then assigned to these observations accordingly: 0–3 (normal flora), 4–6 (intermediate or mixed flora), and 7–10 (BV).^11,12^

**Figure 3:**
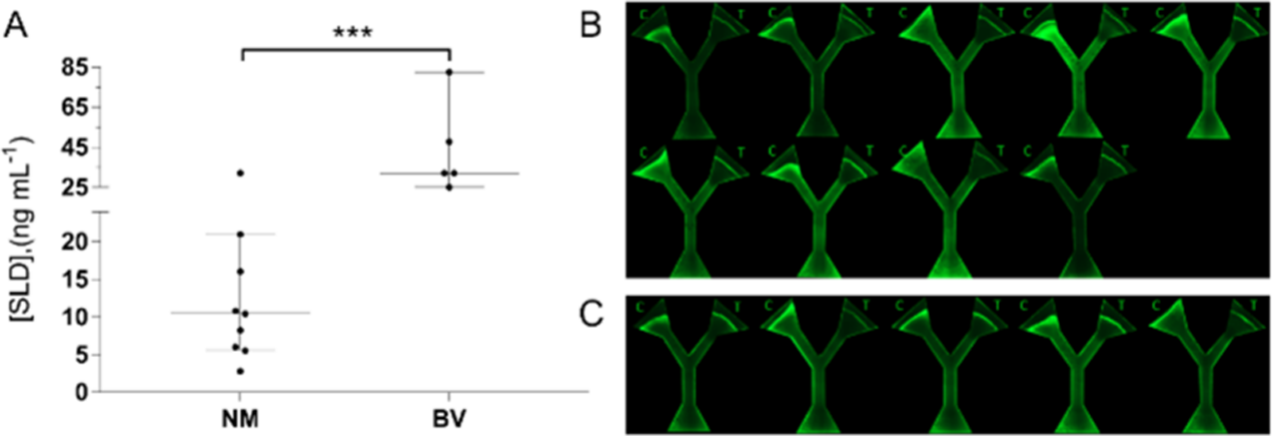
Analysis of clinical samples (vaginal swabs), dilution factor 1:32. Nine samples of NM cases and five samples of BV cases were analyzed. (A) Distribution of the analyzed groups. (B) Fluorescence micrographs of the NM samples tested. (C) Fluorescence micrographs of the BV samples tested.

Figure 3A shows the analytical behavior of the analyzed clinical samples. In general, the samples from NM patients exhibited a concentration lower than 25.1 ng mL^–1^, and the samples from BV patients displayed a concentration higher than 25.1 ng mL^–1^, which is consistent with an immunoassay reported previously.^11^ Likewise, low SLD concentrations (<25.1 ng mL^–1^) are related to those samples with a 0–3 Nugent score and 1–2 Amsel criteria, whereas high SLD concentrations (>25.1 ng mL^–1^) correspond to a 7–10 Nugent score and more than 2 Amsel criteria (see Tables S2 and S3). It is noteworthy that there is only one NM sample overlapping the BV group, which could be considered a false-positive case within the explored samples; however, due to the limited availability of clinical samples (*n* = 14), a robust determination of the respective clinical sensitivity or specificity of the developed device is out of the scope of this report. Figure 3B,C shows the micrographs corresponding to the analyzed samples, NM and BV respectively. The CV (resulting from three parallel assays) of those disposable devices tested with NM samples ranged from 3.14 to 14.75%, whereas the CV of those disposable devices tested with BV samples ranged from 3.48 to 11.31% (see Table S6 in the Supporting Information). Consequently, these ranges of CV meet the maximal variability recommended for clinical analysis, which is around 15%.^28^ Importantly, given the filtration capabilities of paper-based devices,^29^ the vaginal swabs specimens were not centrifuged prior to analysis via the proposed disposable device. Therefore, our device also simplified the immunoassay procedure in terms of sample preparation, especially when compared with our previously reported nanophotonic immunoassay, which required a centrifugation step.^11^

## Conclusions

Exploiting an immunosensing technology that only requires a single antibody to capture and detect the analyte,^11,19−21^ we engineered a low-cost disposable device with potential application at the POC. The proposed disposable device has been optimized to achieve a sensitive and fast response for BV detection (within 20 min). At the laboratory scale, the disposable device had an estimated cost of 1.85 USD (see details in Table S7 in the Supporting Information). As future work, we aim to develop a paper-based fluorescence reader designed for FITC,^30^ which will allow for the employment of the disposable device at the POC. Using Amsel and Nugent criteria as conventional methods for BV diagnosis, the device was proven useful to analyze clinical samples and discriminate between NM and BV cases. The disposable device can be applied in (1) the timely detection of BV, (2) BV therapy monitoring by evaluating SLD levels across time, and (3) the detection asymptomatic cases of BV and prevent its consequences. In addition, the proposed device is highly transformative and can be employed in the detection of other analytes by simply changing the biorecognition element linked to a fluorophore.

## Supporting Information Available

Optimization of the disposable device, resulting CVs, comparison of the analytical results with Nugent and Amsel criteria, and estimation of the cost (PDF)

## Abbreviations

GO: graphene oxide
FAS: FITC-anti sialidase
BV: bacterial vaginosis
NM: normal microbiota
SLD: sialidase

## References

[ref1] Dincer C.; Bruch R.; Costa-Rama E.; Fernández-Abedul M. T.; Merkoçi A.; Manz A.; Urban G. A.; Güder F. Disposable Sensors in Diagnostics, Food, and Environmental Monitoring. Adv. Mater. 2019, 31, 1806739 10.1002/adma.201806739. 31094032

[ref2] Noviana E.; Ozer T.; Carrell C. S.; Link J. S.; McMahon C.; Jang I.; Henry C. S. Microfluidic Paper-Based Analytical Devices: From Design to Applications. Chem. Rev. 2021, 121, 11835–11885. 10.1021/acs.chemrev.0c01335. 34125526

[ref3] Koivunen R.; Jutila E.; Bollström R.; Gane P. Hydrophobic Patterning of Functional Porous Pigment Coatings by Inkjet Printing. Microfluid. Nanofluid. 2016, 20, 83 10.1007/s10404-016-1747-9.

[ref4] Loo J. F. C.; Ho A. H. P.; Turner A. P. F.; Mak W. C. Integrated Printed Microfluidic Biosensors. Trends Biotechnol. 2019, 37, 1104–1120. 10.1016/j.tibtech.2019.03.009. 30992149

[ref5] Reda A.; El-Safty S. A.; Selim M. M.; Shenashen M. A. Optical Glucose Biosensor Built-in Disposable Strips and Wearable Electronic Devices. Biosens. Bioelectron. 2021, 185, 113237 10.1016/j.bios.2021.113237. 33932881

[ref6] Feng L.-X.; Tang C.; Han X.-X.; Zhang H.-C.; Guo F.-N.; Yang T.; Wang J.-H. Simultaneous and Sensitive Detection of Multiple Small Biological Molecules by Microfluidic Paper-Based Analytical Device Integrated with Zinc Oxide Nanorods. Talanta 2021, 232, 122499 10.1016/j.talanta.2021.122499. 34074451

[ref7] Ng A. H. C.; Fobel R.; Fobel C.; Lamanna J.; Rackus D. G.; Summers A.; Dixon C.; Dryden M. D. M.; Lam C.; Ho M.; Mufti N. S.; Lee V.; Asri M. A. M.; Sykes E. A.; Chamberlain M. D.; Joseph R.; Ope M.; Scobie H. M.; Knipes A.; Rota P. A.; Marano N.; Chege P. M.; Njuguna M.; Nzunza R.; Kisangau N.; Kiogora J.; Karuingi M.; Burton J. W.; Borus P.; Lam E.; Wheeler A. R. A Digital Microfluidic System for Serological Immunoassays in Remote Settings. Sci. Transl. Med. 2018, 10, eaar6076 10.1126/scitranslmed.aar6076. 29695457

[ref8] Lin D.; Li B.; Fu L.; Qi J.; Xia C.; Zhang Y.; Chen J.; Choo J.; Chen L. A Novel Polymer-Based Nitrocellulose Platform for Implementing a Multiplexed Microfluidic Paper-Based Enzyme-Linked Immunosorbent Assay. Microsyst. Nanoeng. 2022, 8, 53 10.1038/s41378-022-00385-z. 35600221 PMC9120459

[ref9] Kaarj K.; Akarapipad P.; Yoon J.-Y. Simpler, Faster, and Sensitive Zika Virus Assay Using Smartphone Detection of Loop-Mediated Isothermal Amplification on Paper Microfluidic Chips. Sci. Rep. 2018, 8, 12438 10.1038/s41598-018-30797-9. 30127503 PMC6102244

[ref10] Zhao Y.; Zeng D.; Yan C.; Chen W.; Ren J.; Jiang Y.; Jiang L.; Xue F.; Ji D.; Tang F.; Zhou M.; Dai J. Rapid and Accurate Detection of Escherichia Coli O157:H7 in Beef Using Microfluidic Wax-Printed Paper-Based ELISA. Analyst 2020, 145, 3106–3115. 10.1039/d0an00224k. 32159201

[ref11] Rodríguez-Nava C.; Cortés-Sarabia K.; Avila-Huerta M. D.; Ortiz-Riaño E. J.; Estrada-Moreno A. K.; Alarcón-Romero L.; Mata-Ruíz O.; Medina-Flores Y.; Vences-Velázquez A.; Morales-Narváez E. Nanophotonic Sialidase Immunoassay for Bacterial Vaginosis Diagnosis. ACS Pharmacol. Transl. Sci. 2021, 4, 365–371. 10.1021/acsptsci.0c00211. 33615186 PMC7887842

[ref12] Coleman J. S.; Gaydos C. A. Molecular Diagnosis of Bacterial Vaginosis: An Update. J. Clin. Microbiol. 2018, 56, e00342–18. 10.1128/jcm.00342-18. 29769280 PMC6113459

[ref13] Mahajan G.; Doherty E.; To T.; Sutherland A.; Grant J.; Junaid A.; Gulati A.; LoGrande N.; Izadifar Z.; Timilsina S. S.; Horváth V.; Plebani R.; France M.; Hood-Pishchany I.; Rakoff-Nahoum S.; Kwon D. S.; Goyal G.; Prantil-Baun R.; Ravel J.; Ingber D. E. Vaginal Microbiome-Host Interactions Modeled in a Human Vagina-on-a-Chip. Microbiome 2022, 10, 201 10.1186/s40168-022-01400-1. 36434666 PMC9701078

[ref14] Amsel R.; Totten P. A.; Spiegel C. A.; Chen K. C. S.; Eschenbach D.; Holmes K. K. Nonspecific Vaginitis: Diagnostic Criteria and Microbial and Epidemiologic Associations. Am. J. Med. 1983, 74, 14–22. 10.1016/0002-9343(83)91112-9. 6600371

[ref15] Nugent R. P.; Krohn M. A.; Hillier S. L. Reliability of Diagnosing Bacterial Vaginosis Is Improved by a Standardized Method of Gram Stain Interpretation. J. Clin. Microbiol. 1991, 29, 297–301. 10.1128/jcm.29.2.297-301.1991. 1706728 PMC269757

[ref16] Redelinghuys M. J.; Geldenhuys J.; Jung H.; Kock M. M. Bacterial Vaginosis: Current Diagnostic Avenues and Future Opportunities. Front. Cell. Infect. Microbiol. 2020, 10, 354 10.3389/fcimb.2020.00354. 32850469 PMC7431474

[ref17] Sekisui Diagnostics. OSOM BVBLUE Test. https://sekisuidiagnostics.com/product/osom-bvblue-test/ (accessed April 17, 2023).

[ref18] Cortés-Sarabia K.; Rodríguez-Nava C.; Medina-Flores Y.; Mata-Ruíz O.; López-Meza J. E.; Gómez-Cervantes M. D.; Parra-Rojas I.; Illades-Aguiar B.; Flores-Alfaro E.; Vences-Velázquez A. Production and Characterization of a Monoclonal Antibody against the Sialidase of Gardnerella Vaginalis Using a Synthetic Peptide in a MAP8 Format. Appl. Microbiol. Biotechnol. 2020, 104, 6173–6183. 10.1007/s00253-020-10691-z. 32462244 PMC7253150

[ref19] Avila-Huerta M. D.; Ortiz-Riaño E. J.; Mancera-Zapata D. L.; Morales-Narváez E. Real-Time Photoluminescent Biosensing Based on Graphene Oxide-Coated Microplates: A Rapid Pathogen Detection Platform. Anal. Chem. 2020, 92, 11511–11515. 10.1021/acs.analchem.0c02200. 32603091

[ref20] Ortiz-Riaño E. J.; Avila-Huerta M. D.; Mancera-Zapata D. L.; Morales-Narváez E. Microwell Plates Coated with Graphene Oxide Enable Advantageous Real-Time Immunosensing Platform. Biosens. Bioelectron. 2020, 165, 112319 10.1016/j.bios.2020.112319. 32729472

[ref21] Avila-Huerta M. D.; Ortiz-Riaño E. J.; Mancera-Zapata D. L.; Cortés-Sarabia K.; Morales-Narváez E. Facile Determination of COVID-19 Seroconversion via Nonradiative Energy Transfer. ACS Sens. 2021, 6, 2136–2140. 10.1021/acssensors.1c00795. 34047541

[ref22] Morales-Narváez E.; Merkoçi A. Graphene Oxide as an Optical Biosensing Platform: A Progress Report. Adv. Mater. 2019, 31, 1805043 10.1002/adma.201805043. 30549101

[ref23] Imani M.; Mohajeri N.; Rastegar M.; Zarghami N. Recent Advances in FRET-Based Biosensors for Biomedical Applications. Anal. Biochem. 2021, 630, 114323 10.1016/j.ab.2021.114323. 34339665

[ref24] Tan Y. H.; Liu M.; Nolting B.; Go J. G.; Gervay-Hague J.; Liu G. A Nanoengineering Approach for Investigation and Regulation of Protein Immobilization. ACS Nano 2008, 2, 2374–2384. 10.1021/nn800508f. 19206405 PMC4512660

[ref25] Robinson L. S.; Schwebke J.; Lewis W. G.; Lewis A. L. Identification and Characterization of NanH2 and NanH3, Enzymes Responsible for Sialidase Activity in the Vaginal Bacterium Gardnerella Vaginalis. J. Biol. Chem. 2019, 294, 5230–5245. 10.1074/jbc.ra118.006221. 30723162 PMC6462536

[ref26] Tang R.; Xie M. Y.; Li M.; Cao L.; Feng S.; Li Z.; Xu F. Nitrocellulose Membrane for Paper-Based Biosensor. Appl. Mater. Today 2022, 26, 101305 10.1016/j.apmt.2021.101305.

[ref27] Ramirez-Priego P.; Estévez M.-C.; Díaz-Luisravelo H. J.; Manclús J. J.; Montoya Á.; Lechuga L. M. Real-Time Monitoring of Fenitrothion in Water Samples Using a Silicon Nanophotonic Biosensor. Anal. Chim. Acta 2021, 1152, 338276 10.1016/j.aca.2021.338276. 33648644

[ref28] Peláez E. C.; Estevez M. C.; Mongui A.; Menéndez M.-C.; Toro C.; Herrera-Sandoval O. L.; Robledo J.; García M. J.; Portillo P. D.; Lechuga L. M. Detection and Quantification of HspX Antigen in Sputum Samples Using Plasmonic Biosensing: Toward a Real Point-of-Care (POC) for Tuberculosis Diagnosis. ACS Infect. Dis. 2020, 6, 1110–1120. 10.1021/acsinfecdis.9b00502. 32233503

[ref29] Sui J.; Lin H.; Xu Y.; Cao L. Enhancement of Dot-Immunogold Filtration Assay (DIGFA) by Activation of Nitrocellulose Membranes with Secondary Antibody. Food Anal. Methods 2011, 4, 245–250. 10.1007/s12161-010-9137-5.

[ref30] Ireta-Muñoz L. A.; Morales-Narváez E. Smartphone and Paper-Based Fluorescence Reader: A Do It Yourself Approach. Biosensors 2020, 10, 60 10.3390/bios10060060. 32498366 PMC7345677

