# Supplementary material for: Disposable Device for Bacterial Vaginosis Detection
Source: ACS Meas Sci Au. 2023 Jun 17;3(5):355–60. doi: 10.1021/acsmeasuresciau.3c00007 (PMC10588930; doi:10.1021/acsmeasuresciau.3c00007)
Supplement: Supplementary file 1 — tg3c00007_si_001.pdf [file tg3c00007_si_001.pdf]

Supporting Information

Disposable device for Bacterial Vaginosis Detection

Mariana D. Avila-Huerta[1], Karina Leyva-Hidalgo[1,2], Karen Cortés-Sarabia[2], Ana K. Estrada-Moreno[2], Amalia Vences-Velázquez[2], Eden Morales-Narváez[1,3*]

[1] Centro de Investigaciones en Óptica, A. C., Loma del Bosque 115, Lomas del Campestre, León 37150, Guanajuato, Mexico

[2] Facultad de Ciencias Químico Biológicas, Universidad Autónoma de Guerrero, Chilpancingo 39070, Guerrero, Mexico

[3] Biophotonic Nanosensors Laboratory, Centro de Física Aplicada y Tecnología Avanzada (CFATA), Universidad Nacional Autónoma de México (UNAM), Querétaro, 76230, México

[*] eden@fata.unam.mx

Table of Contents

Figure S1. Estimation of the distance between FITC and GO.

Figure S2. Optimization of the channel width.

Figure S3. First design of the disposable device.

Figure S4. Optimization of the concentration of Tween 20

Figure S5. Optimization of the design of the microfluidic circuit.

Figure S6. Optimal design of the microfluidic circuit.

Figure S7. FAS and GO optimization.

Figure S8. Model depicting the employed image analysis method.

Figure S9. Quantitative performance of the proposed disposable device.

Figure S10. Evaluation of different dilution factors of the clinical samples.

Table S1. Optimization design of microfluidic circuit

Table S2. NM samples. Comparison of Amsel/Nugent citeria and the concentration measured though the proposed device.

Table S3. BV samples. Comparison of Amsel/Nugent citeria and the concentration measured though the proposed device.

Table S4. The resulting p values (corresponding to a t-test) to determine the optimal dilution factor of the vaginal swab samples.

Table S5. CVs of the disposable biosensor using [FAS]=80 μg mL $^{-1}$.

Table S6. CVs of the disposable biosensor testing clinical samples.

Table S7. Estimation of the cost of the developed device.

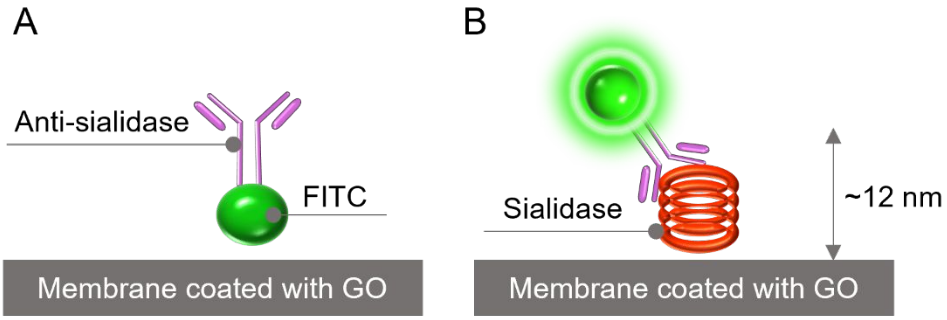

Figure S1. Estimated distance between FITC and GO. The distance between the FITC (from FAS) and the GO (in the surface of the membrane) can be estimated considering the size of the anti-sialidase antibody plus the size of sialidase, which is 8.4 nm plus 4 nm, respectively, that is, c.a. 12.4 nm. A) When sialidase is absent, the distance between FAS and the membrane coated with GO is minimum, which allows a strong energy transfer phenomenon. B) In the presence sialidase, an immunocomplex is formed and the estimated distance between the GO-coated nitrocellulose and FAS is about 12 nm, which avoids strong energy transfer between FITC (donor) and GO (acceptor).

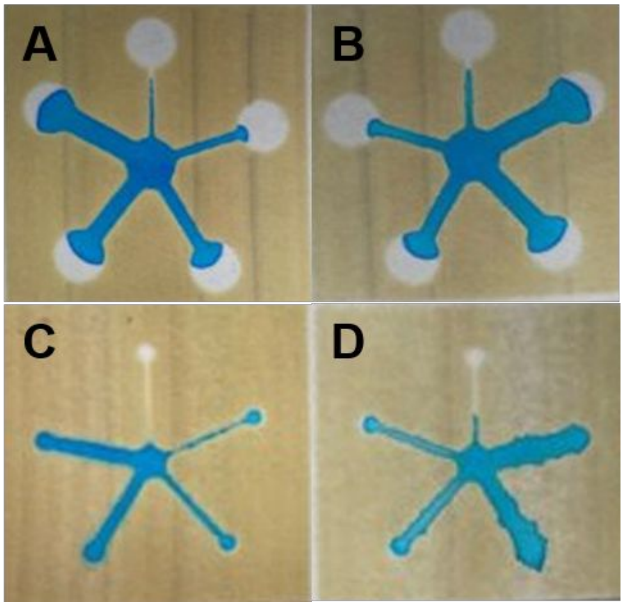

Figure S2. Optimization of the channel width. **A**. Front side of the device. Channel width assessment, from 1 to 3 mm (1, 1.5, 2, 2.5,3 mm). **B**. Back side of the device. Channel width evaluation, from 1 to 3 mm. **C**. Front side of the device. Channel width optimization, from 1 to 1.5 mm (0.6, 0.8, 1, 1.3, 1.5 mm). **D**. Back side of the device. Channel width evaluation, from 1 to 1.5 mm

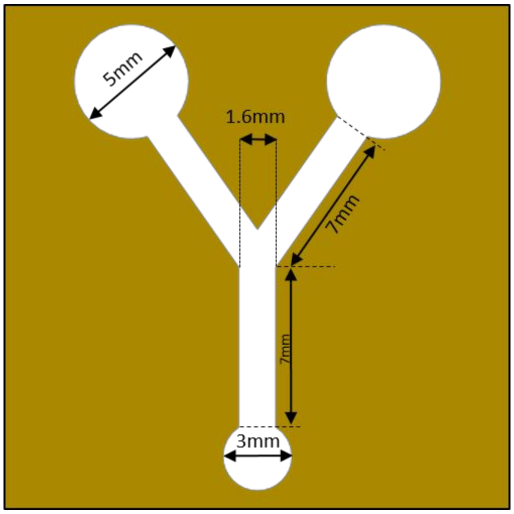

Figure S3. First design of the disposable device.

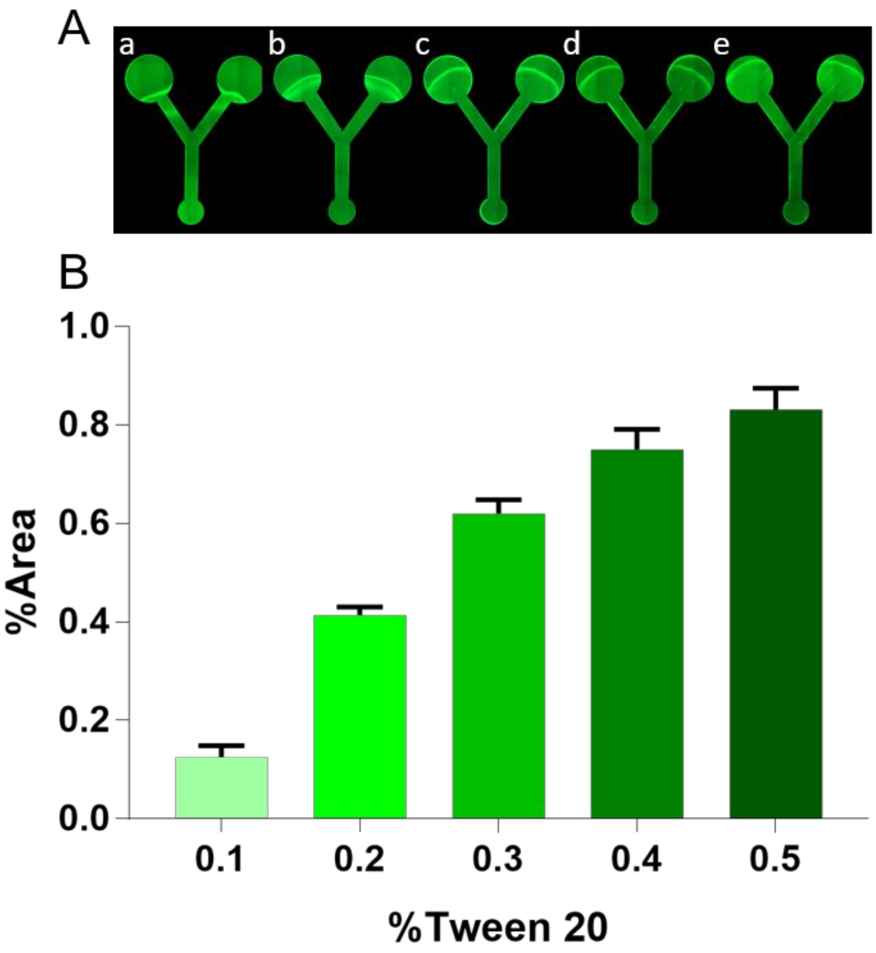

Figure S4. Optimization of the concentration of Tween 20 (concentration of the fluorescent probe, [FAS] = 10 ug mL-1). **A**. Fluorescence microscope images of the µPAD using a) 0.1%, b) 0.2%, c) 0.3%, d) 0.4%, e) 0.5% of Tween 20. **B**. Percentage of the area covered by the sample with different concentrations of Tween 20.

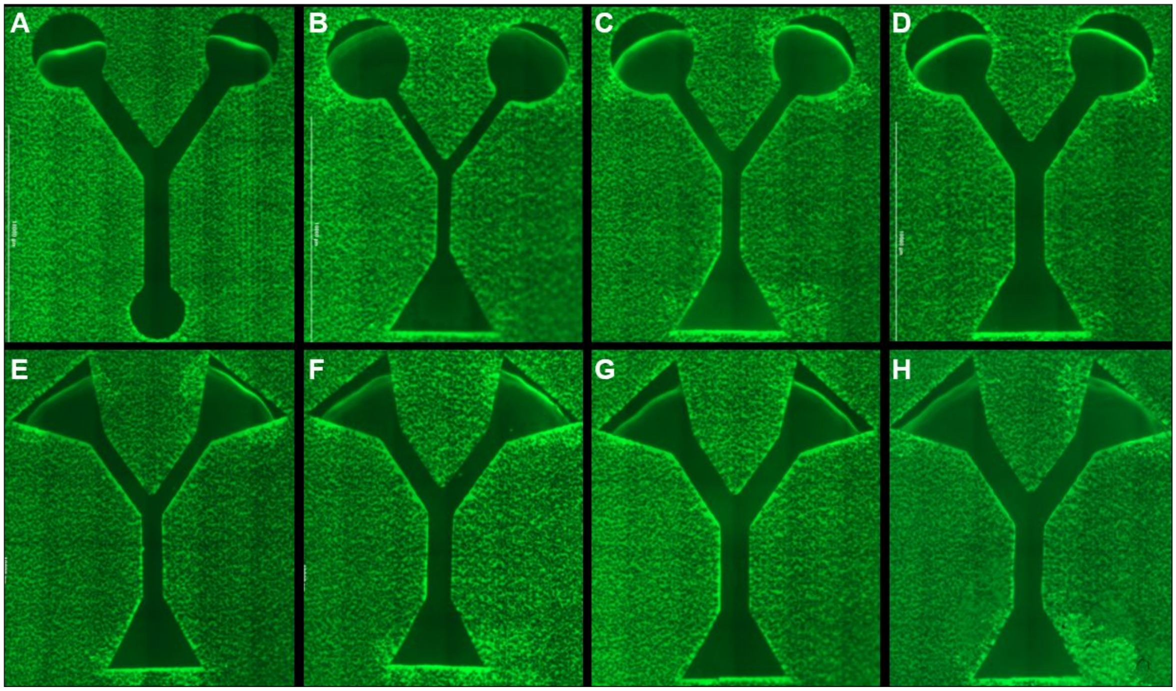

Figure S5. Optimization of the design of the microfluidic circuit. See Table S1, each image corresponds to a row of such a Table.

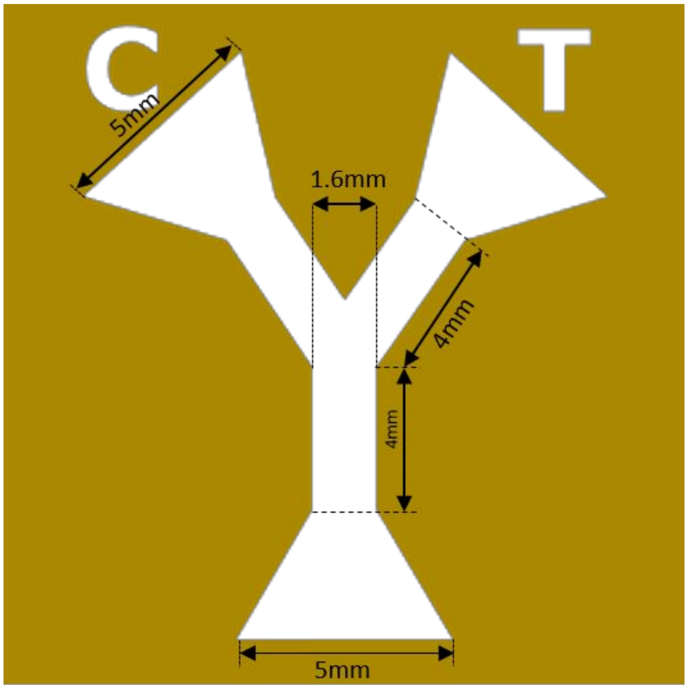

Figure S6. Optimal design of the microfluidic circuit.

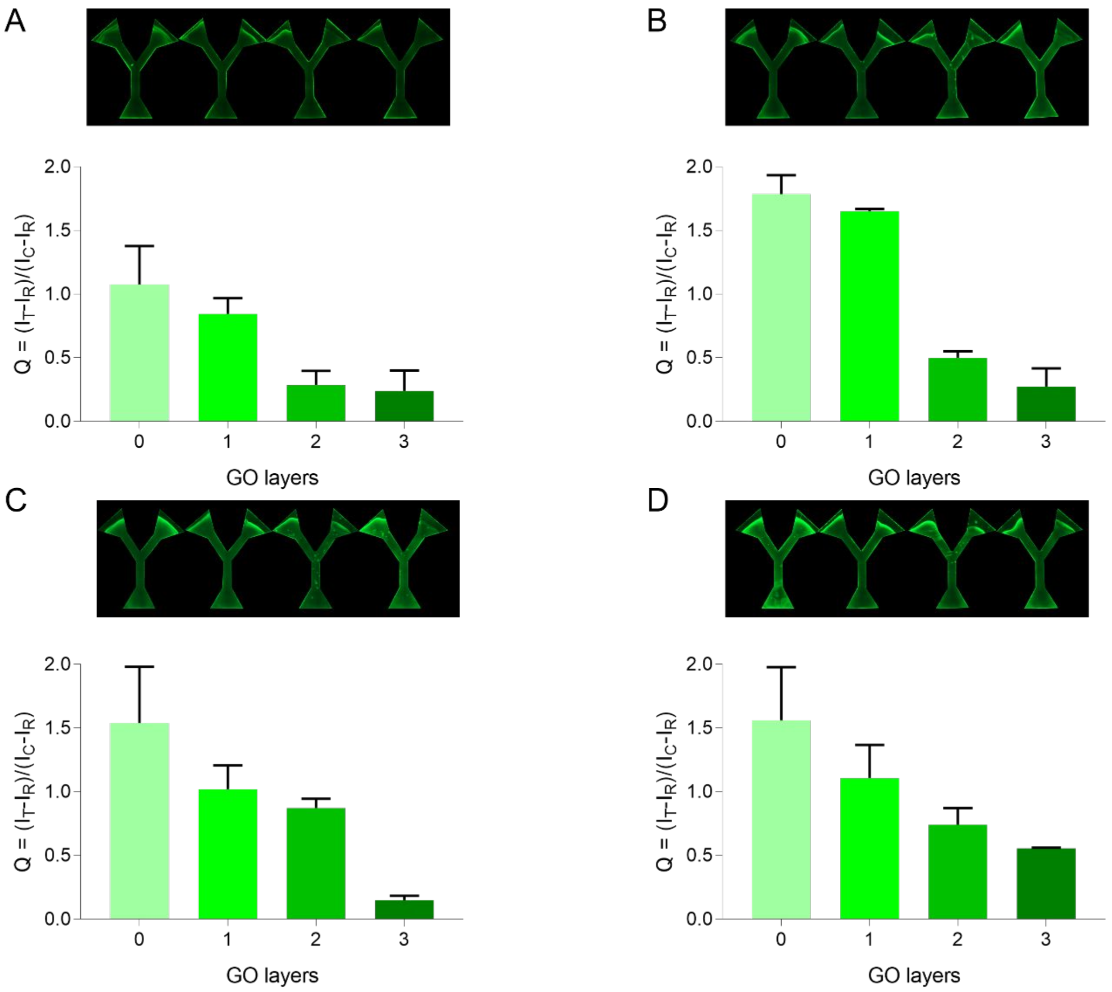

Figure S7. FAS and GO optimization. 0, 1, 2 and 3 layers of 5µL of GO concentrated at 500 µg mL^{-1} were used for each FAS concentration explored. **A**. Evaluation of the fluorescence quenching in terms of the *Q* ratio (see Eq. 1) in those devices tested with [FAS] = 40 µg mL^{-1}. **B**. *Q* ratio in those devices tested with [FAS] = 60µg mL^{-1}. **C**. *Q* ratio in the devices tested with [FAS] = 80µg mL^{-1}. **D**. Q ratio in those devices tested with [FAS] = 100µg mL^{-1}.

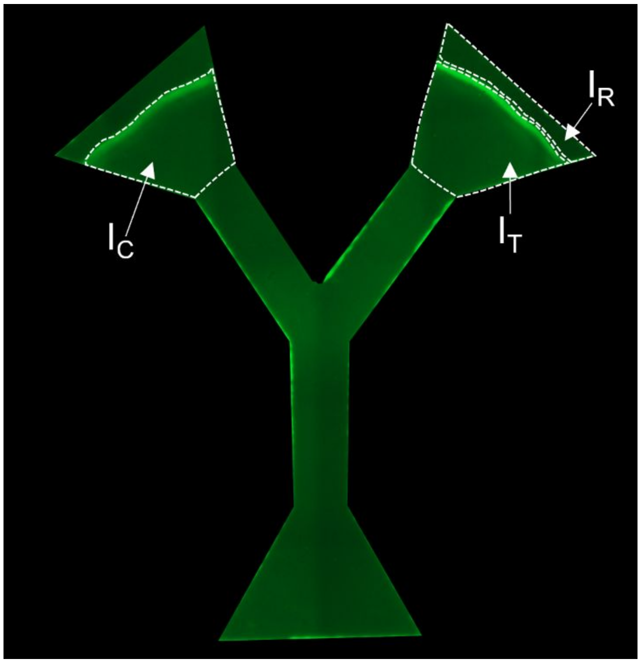

Figure S8. Model depicting the employed image analysis method. IC: Average intensity of the pixels in the control area. IT: Average intensity of the pixels in the test area. IR: Average intensity of the pixels in the background area.

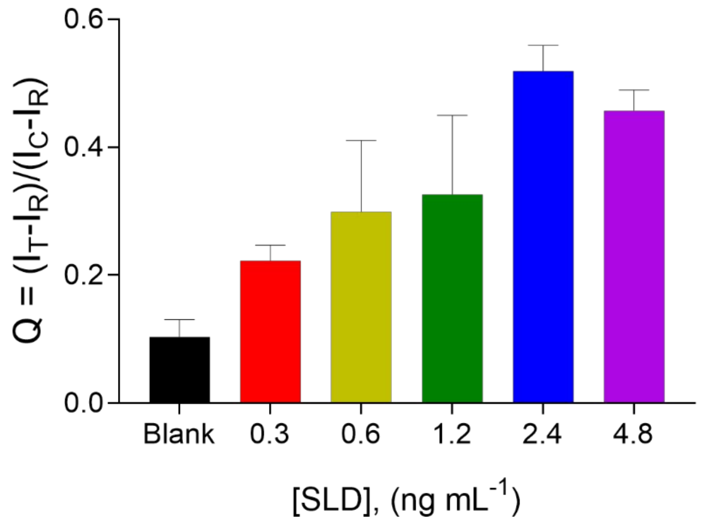

Figure S9. Quantitative performance of the proposed disposable device. FAS was employed at 80 μg mL^{-1} and 3 layers of GO concentrated at 500 μg mL^{-1} were deposited in the corresponding device. Dynamic range from 0.3 to 4.8 ng mL^{-1}.

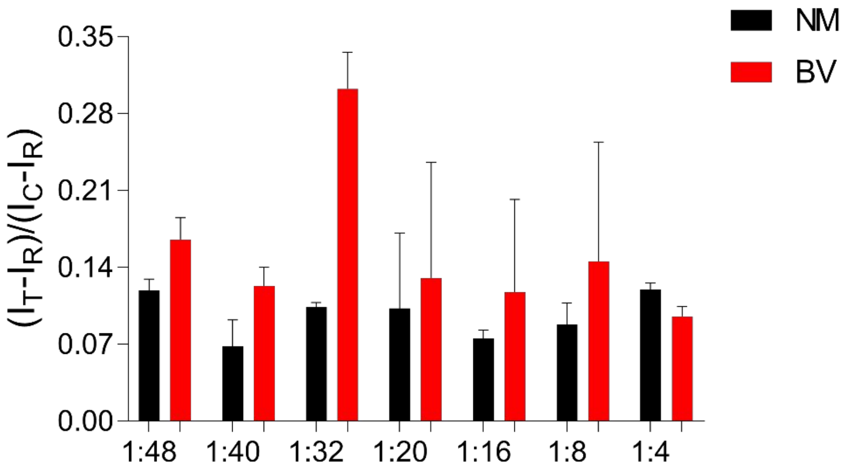

Figure S10. Evaluation of different dilution factors of the clinical samples. NM, normal microbiota sample. BV, bacterial vaginosis sample.

Table S1. Optimization of the design of the microfluidic circuit.

#	Entrance geometry	Entrance width	Trunk width	Trunk length	Branches width	Branches length	Control/Test geometry	Control/Test width	Volume (µL)	Outflow	Covered area
A	circle	5	1.6	7	1.6	7	circle	4	5	NO	65.18%
B	trapeze	5	1	4	1	4	circle	4	6	YES	85.35%
C	trapeze	5	1.2	4	1.2	4	circle	4	6	YES	75.86%
D	trapeze	5	1.6	4	1.6	4	circle	4	6	YES	65.81%
E	trapeze	5	1.2	4	1.2	4	trapeze	5	6	YES	76.60%
F	trapeze	5	1.4	4	1.4	4	trapeze	5	6	YES	79.99%
**G**	**trapeze**	**5**	**1.6**	**4**	**1.6**	**4**	**trapeze**	**5**	**6**	**NO**	**75.22%**
H	trapeze	5	1.6	4	1.6	4	trapeze	5	7	YES	84.04%

Table S2. NM samples. Comparison of Amsel/Nugent criteria and the concentration measured though the proposed device.

Sample	Nugent score	Amsel Diagnostic	μPAD (Q ratio)	MC μPAD (ng mL^{-1})
NM 1	0-3	1-2	0.199 ± 0.0147	6.016
NM 2	0-3	1-2	0.283 ± 0.023	20.992
NM 3	0-3	3-4	0.194 ± 0.0248	5.548
NM 4	0-3	1-2	0.158 ± 0.0042	2.808
NM 5	0-3	1-2	0.218 ± 0.0100	8.216
NM 6	0-3	0	0.262 ± 0.0158	16.067
NM 7	0-3	0	0.329 ± 0.0486	32.116
NM 8	0-3	1-2	0.232 ± 0.0148	10.433
NM 9	0-3	0	0.235 ± 0.0257	10.823

*MC μPAD: Concentration measured with the μPAD biosensor

Table S3. BV samples. Comparison of Amsel/Nugent criteria and the concentration measured though the proposed device.

Sample	Nugent score	Amsel Diagnostic	μPAD (Q ratio)	MC μPAD (ng mL^{-1})
BV 1	7-10	3 - 4	0.329 ± 0.026	32.086
BV 2	7-10	3 - 4	0.300 ± 0.0111	25.117
BV 3	7-10	1-2	0.534 ± 0.0186	82.460
BV 4	7-10	unrealized	0.393 ± 0.0351	47.742
BV 5	7-10	1 – 2	0.329 ± 0.0271	32.130

*MC μPAD: Measured concentration with the μPAD biosensor

Table S4. The resulting p values (corresponding to a t-test) to determine the optimal dilution factor of the vaginal swab samples.

Dilution	p
1:4	0.0311
1:8	0.4196
1:16	0.4370
1:20	0.7697
**1:32**	**0.0141**
1:40	0.0724
1:48	0.0246

Table S5. CVs of the disposable biosensor testing standard samples.

[Sialidase], (ng mL^{-1})	CV
0	4.91%
0.3	11.05%
0.6	14.69%
1.2	7.31%
2.4	7.69%
4.8	7.12%

Table S6. CVs of the disposable biosensor testing clinical samples.

Sample	CV
NM 1	11.33%
NM 2	8.13%
NM 3	13.59%
NM 4	3.14%
NM 5	4.82%
NM 6	6.02%
NM 7	14.75%
NM 8	4.59%
NM 9	9.78%
BV 1	10.90%
BV 2	11.31%
BV 3	3.48%
BV 4	9.99%
BV 5	7.49%

Table S7. Estimation of the cost of the disposable device.

	Total volume	Concentration	Cost	Volume per device	Required concentration	Cost per device
Nitrocellulose	100 m	N/A	$ 290.00	0.025 m	N/A	$ 0.0725
Wax	264000 devices	N/A	$ 130.00	1 device	N/A	$ 0.0005
GO	1 L	5000 µg mL^{-1}	$ 210.00	1.5 µL	500 µg mL^{-1}	$ 0.0003
FAS	120 µL	833.33 µg mL^{-1}	$ 375.00	0.57 µL	160 µg mL^{-1}	$ 1.78
PBS	100 tablets	N/A	$ 130.00	6 µL	N/A	$ 0.0002
Tween 20	1 L	N/A	$ 100.00	0.03 µL	N/A	$ 0.000003
					**Total (per device)**	**$ 1.8548**

*FAS: Antibody conjugated with FITC. The cost of 100µL of [Anti-sialidase] = 1 mg mL^{-1} is $55.00 USD. The cost of FITC conjugation kit is $320.00 USD.
